# Down-regulation of a *LBD*-like gene, *OsIG1*, leads to occurrence of unusual double ovules and developmental abnormalities of various floral organs and megagametophyte in rice

**DOI:** 10.1093/jxb/eru396

**Published:** 2014-10-16

**Authors:** Jingrong Zhang, Wei Tang, Yulan Huang, Xiangli Niu, Yu Zhao, Yi Han, Yongsheng Liu

**Affiliations:** ^1^Ministry of Education Key Laboratory for Bio-resource and Eco-environment, College of Life Science, State Key Laboratory of Hydraulics and Mountain River Engineering, Sichuan University, Chengdu 610064, China; ^2^School of Biotechnology and Food Engineering, Hefei University of Technology, Hefei 230009, China; ^3^National Key Laboratory of Crop Genetics and Improvement, Huazhong Agricultural University, Wuhan 430070, China

**Keywords:** embryo sac, empty glume, female gametophyte, *OsIG1*, ovule.

## Abstract

The *indeterminate gametophyte1* (*ig1*) mutation was first characterized to modulate female gametophyte development in maize (*Zea mays*). However, the function of its rice orthologue, *OsIG1*, remains unknown. For this, we first analysed *OsIG1* localization from differential tissues in rice. Real-time quantitative PCR (qRT-PCR) and histochemical staining results demonstrated that the expression signal of *OsIG1* was strongly detected in young inflorescence, moderately in mature flower and weakly in leaf. Furthermore, RNA *in situ* hybridization analyses exhibited that *OsIG1* was strongly expressed in inflorescence meristems, floral meristems, empty-glume- and floret- primordia, especially in the primordia of stamens and immature ovules, and the micropylar side of the mature ovary. In *OsIG1*-RNAi lines, wrinkled blade formation was accompanied by increased leaf inclination angle. Cross-section further showed that the number of bulliform cells located between the vasculatures was significantly increased, indicating that *OsIG1* is involved in division and differentiation of bulliform cell and lateral growth during leaf development. *OsIG1*-RNAi suppression lines showed pleiotropic phenotypes, including degenerated palea, glume-like features and open hull. In addition, a single *OsIG1-*RNAi floret is characterized by frequently developing double ovules with abnormal embryo sac development. Additionally, down-regulation of *OsIG1* differentially affected the expression of genes associated with the floral organ development including *EG1*, *OsMADS6* and *OsMADS1*. Taken together, these results demonstrate that *OsIG1* plays an essential role in the regulation of empty-glume identity, floral organ number control and female gametophyte development in rice.

## Highlight

This study demonstrates that OsIG1 plays an essential role in the regulation of empty-glume identity, floral organ number control and female gametophyte development in rice.

## Introduction

The LATERAL ORGAN BOUNDARIES DOMAIN/ASYMMETRIC LEAVES2-LIKE (LBD/ASL) proteins (hereafter referred to as LBD) were identified as plant-specific transcription factors harbouring a conserved LATERAL ORGAN BOUNDARIES (LOB) domain (Ori *et al.*, 2000; Semiarti *et al.*, 2001; Chalfun-Junior *et al.*, 2005; Borghi *et al.*, 2007). The LOB domain includes a zinc-finger motif responsible for DNA binding and a leucine-zipper-like motif mediating protein–protein interactions (Husbands *et al.*, 2007). The LBD family comprises 43 members in *Arabidopsis* (Iwakawa *et al.*, 2002; Shuai *et al.*, 2002), 35 members in rice (*Oryza sativa*; Yang *et al.*, 2006), and 57 in poplar (Zhu *et al.*, 2007), respectively. Previous studies indicated that LBD proteins play a vital role in determining of leaf adaxial–abaxial polarity in *Arabidopsis* and maize (Lin *et al.*, 2003; Xu *et al.*, 2003; Evans, 2007) through repressing the expression of *KNOTTED-LIKE HOMEOBOX* (*KNOX*) genes in lateral organs (Ori *et al.*, 2000; Semiarti *et al.*, 2001; Chalfun-Junior *et al.*, 2005; Borghi *et al.*, 2007; Evans, 2007). Additionally, several *LBD* genes, such as *RTCS* in maize and *Crown rootless1* in rice, were implicated in the modulation of crown and lateral root development in an auxin-dependent pathway (Inukai *et al.*, 2005; Liu *et al.*, 2005; Okushima *et al.*, 2007; Lee *et al.*, 2009).

It has also been demonstrated that LBD proteins are involved in reproductive organogenesis as well as vegetative growth (Xu *et al.*, 2003; Evans, 2007). For instance, in maize, the *ramosa2* gene containing an LOB domain was revealed to determine inflorescence branch formation (Bortiri *et al.*, 2006). The maize *indeterminate gametophyte1* (*ig1*) gene was found to regulate female gametophyte development (Evans, 2007). The *ig1* mutant fails to restrict the proliferative phase in the embryo sac, resulting in an indeterminate number of eggs, polar nuclei and synergids (Evans, 2007). The rice *LBD* gene *degenerated hull1* (*DH1*) is required for glume formation, as demonstrated by the phenotype of its T-DNA insert mutant with degenerated hull and naked pistils and stamens (Li *et al.*, 2008). Despite substantial investigations, little is known about the links between LBD proteins and floral organ development.

In angiosperms, the ovule is located within the pistil, which consists of one or more carpels. As the female reproductive structures, ovules are eventually developed into seeds to generate new population of offspring. By far, only a few genes which regulate ovule and carpel development have been characterized in plants (Lopez-Dee *et al.*, 1999; Pagnussat *et al.*, 2004; Skinner *et al.*, 2004). Most of the isolated ovule identity genes belong to MADS-box genes. In *Arabidopsis thaliana*, cell fate in proliferating ovule primordia is specified by the MADS box gene *STK* (*SEEDSTICK*; Pinyopich *et al.*, 2003). In rice, a loss-of-function mutant of D-lineage MADS-box gene *OsMADS13* had the ovule replaced with carpelloid structures (Dreni *et al.*, 2007; Yamaki *et al.*, 2011). Both the *YABBY* gene *DROOPING LEAF* (*DL*) and MADS-box gene *OsMADS3* were shown to genetically interact with *OsMADS13* in the regulation of ovule identity and floral meristem determinacy (Li *et al.*, 2011*a*). Furthermore, *OsMADS6* and *OsMADS58* are genetically associated with rice *AGAMOUS* (*AG*) subfamily genes to regulate the identity of stamens, carpels and ovules (Dreni *et al.*, 2011; Li *et al.*, 2011*b*). However, LBD family proteins potentially involved in the regulation of ovule development have not been identified.

Although rice displays a high degree of conserved synteny with maize, it remains unclear whether *LBD* genes are also involved in mediating female gametophyte development and other unknown functions in rice. In this study, we characterized a *LBD* gene *OsIG1* from rice (*O. sativa*), an orthologue of maize *IG1*, and uncovered its novel roles in regulating floral organ development, in addition to its conserved function in female gametophyte development, using genetic and molecular approaches. We further revealed that *OsIG1* might participate in mediating floral development through regulating the expression of some developmentally associated genes including jasmonic acid-synthesizing gene *EG1* (*EXTRA GLUME1*), *AGAMOUS*-*LIKE6*-like gene *OsMADS6* and E-class gene *OsMADS1*. Hence, our results allowed us to infer previously undescribed regulatory roles for *OsIG1* in rice.

## Materials and methods

### Plant materials and growth conditions

Rice transgenic lines in a Nipponbare (*O. sativa* ssp. *japonica*) background and wild-type (WT) Nipponbare were used throughout this study. The plants were grown in farm fields at Sichuan University, Chengdu, China. Primary transformants (T_0_) were first grown in the artificial climate incubators (Binder, Tuttlington, Germany) under standard conditions (28 °C day/20 °C night, 12h light/12h dark) and transplanted into the field 5 weeks later.

### Plasmid construction and rice transformation

To generate an RNAi construct, a 485 base pair (bp) *OsIG1* fragment (*Os01g0889400*; positions 461–810 from the ATG of the coding region plus 135bp of the 3′ untranslated region) was amplified by RT-PCR. An inverted-repeat fragment was constructed into vector pSK int and transferred into the pHB plasmid. To produce the *OsIG1* promoter::GUS fusion construct, the open-reading-frame sequence of β-glucuronidase (*GUS*) gene was obtained from pBI121 by digesting with *Bam*HI and *Sac*I. Then the obtained fragment was inserted into pHB and the resulting construct was denoted GUS-pHB. About 2.1kb of the *OsIG1* gene fragment upstream of the translational start codon was isolated by PCR from Nipponbare genomic DNA (primers are listed in Supplementary Table 1). Products were digested with *Eco*RI and *Bam*HI and ligated into *Eco*RI-/*Bam*HI-digested GUS-pHB to generate plasmid pOsIG1-GUS. The constructs were used to transform Nipponbare callus using engineered *Agrobacterium tumefaciens* (strain EHA101) as described previously (Hiei *et al.*, 1994).

### Tissue preparation and microscopic analyses

Fresh tissues from WT and RNAi plants were analysed with a stereoscopic Leica M420 microscope (Leica Microsystems, Bannockburn, IL, USA) equipped with Leica DFC280 digital camera system. For histological analysis, samples were fixed in FAA solution (3.7% formaldehyde/50% ethanol/5% glacial acetic acid), dehydrated with a series of ethanol solutions, embedded in paraffin and cut into sections 8 µm thick. The sections were stained with ferrovanadium hematoxylin. To evaluate the viability of *OsIG1*-RNAi pollen grains, anthers from transgenic mature flowers were dissected in a drop of 1% I_2_/KI solution. The sections and pollen grains stained were photographed using a Leica DFC280 camera. For scanning electron microscopy, samples were fixed in 2.5% glutaraldehyde solution. Fixed samples were dehydrated with graded ethanol series, dried by critical-point drying, sputter-coated with platinum and observed using a scanning electron microscope [JSM-5900LV; SEMTech (STS), North Billerica, MA, USA].

### RT-PCR and real-time quantitative PCR analysis

Total RNAs were isolated using TRIzol reagent (Invitrogen, Carlsbad, CA, USA) followed by treatment with DNase I to digest genomic DNA (TaKaRa, Dalian, China). About 1μg of total RNA from each sample was used for first-strand cDNA synthesis (Toyobo, Tokyo, Japan). Triplicate quantitative assays were performed using the SYBR Green Master Mix (Applied Biosystems, Foster City, CA, USA) with an ABI PRISM Step One Plus real-time PCR system (Applied Biosystems) according to the manufacturer’s instructions. The relative expression level was determined based on the 2^−∆∆*CT*^ method (Livak and Schmittgen, 2001) by using rice *actin* and *ubiquitin* as internal reference genes. Primers used for real-time quantitative PCR (qRT-PCR) analysis are listed in Supplementary Table 1.

### *In situ* hybridization and histochemical staining

The hybridization and immunological detection were performed as described by Dai *et al* (2007). The gene-specific regions of *OsIG1* were amplified and cloned into pGEM-T Easy (Promega, Madison, WI, USA) for synthesis of an RNA probe; the primers used are shown in Supplementary Table 1. Digoxigenin-labelled sense and antisense probes were synthesized with T7 or SP6 RNA polymerase (Roche, Indianapolis, IN, USA), respectively. GUS staining was performed according to the method described previously (Sieburth and Meyerowitz, 1997).

### Subcellular localization analysis

The coding sequence of the *OsIG1* gene was directionally cloned into the expression vector pART27-mcs:GFP (kindly provided by Professor Shuqing Cao, Hefei University of Technology, Hefei, China), containing the green fluorescent protein (GFP) coding sequence under the control of a 35 S promoter. This generated the pART27-OsIG1-GFP construct. Plasmid vectors pART27-OsIG1-GFP and pART27-GFP were introduced into protoplasts of *Arabidopsis* according to the methods described previously (Yoo *et al.*, 2007). After incubation for 16–20h at 23 °C, protoplasts were observed under a Leica TCS SPII confocal microscope using 488 and 633nm excitation wavelengths and three-channel measurement of emission: 435nm (blue/DAPI), 522nm (green/GFP) and 680nm (red/chlorophyll).

## Results

### The expression pattern of *OsIG1*

According to the previous information (Evans, 2007) and database (http://rapdb.dna.affrc.go.jp/), the genomic sequence of *OsIG1* (*Os01g0889400*) spans 3399bp, and contains three introns and four exons (Supplementary Fig. 1A). It has a deduced coding protein of 270 amino acids with a conserved LOB domain containing C block, GAS block and the predicted coiled-coil structure (Supplementary Fig. 1B; Evans, 2007). Phylogenetic analysis showed that *OsIG1* is closely related to maize *IG1*, and together they are clustered in the same clade with maize *IAL1*, rice *IAL1*, and *Arabidopsis AS2* (Supplementary Fig. 1C; Evans, 2007).

**Fig. 1.**
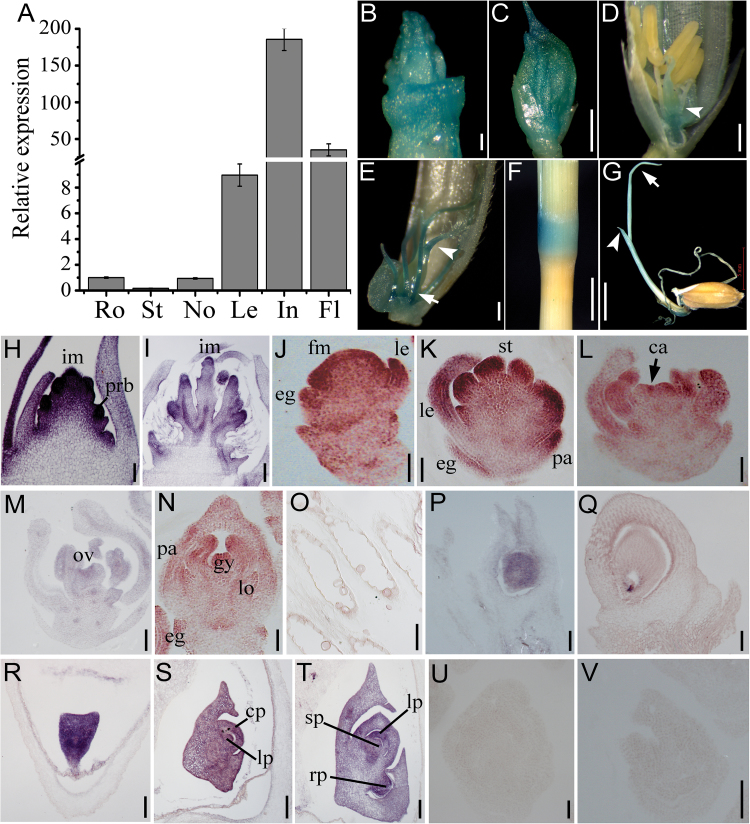
Expression patterns of *OsIG1* in WT of rice. (A) Real-time quantitative RT-PCR analyses of *OsIG1* expressions in various organs of WT rice. RNAs were collected from 3-week-old seedlings: root (Ro), shoot (St), leaf (Le) and inflorescence (In) at stage In5, with the node (No) and flower (Fl) at the mature embryo sac stage. *Actin* was used as a control. The expression of *IG1* in root was set as 1. Error bars represent SD from three independent biological replicates. (B–G) Localization of GUS activity under the control of the *OsIG1* promoter. (B) A 5 mm-long inflorescence; scale bar, 1mm. (C) A 3 mm-long spikelet; bar, 1mm. (D) Postmeiosis spikelet: arrowhead indicates ovary; bar, 200 µm. (E) The mature embryo sac stage: arrow and arrowhead indicate filament and style, respectively; bar, 200 µm. (F) Node; bar, 5mm. (G) Young seedling: arrow and arrowhead indicate primary leaf and second leaf, respectively; bar, 5mm. (H–V) *In situ* hybridization of cross-sections with an *OsIG1*-specifc antisense probe. (H, I) Longitudinal section of a young inflorescence. (J–N) Longitudinal sections of a developing WT spikelet. (O) Anther in nearly mature flowers. (P) Immature ovary. (Q) Mature ovary. (R–T) Sections of WT embryos at 2 days after pollination (DAP) (R), 5 DAP (S) and 7 DAP (T). (U, V) *In situ* analysis using a sense probe of *OsIG1* in the early spikelet as negative controls. ca, carpel; cp, coleoptilar; eg, empty glume; fm, floral meristem; gy, gynoecium; im, inflorescence meristem; le, lemma; lo, lodicule; lp, leaf primordia; ov, ovule; pa, palea; prb, primary rachis branches; rp, root primordium; sp, shoot primordium; st, stamen. Scale bars, 50 μm in (H, J–O, R), 400 μm (I) and 100 μm in others.

To investigate the function of *OsIG1*, we analysed the *OsIG1* expression pattern using qRT-PCR, promoter-GUS fusions and *in situ* hybridization. First, qRT-PCR analysis demonstrated that *OsIG1* was strongly expressed in the young inflorescence, moderately in mature flower and weakly in leaf (Fig. 1A). *OsIG1* expression was lowest in the root and stem relative to other tissues examined (Fig. 1A). To further confirm this result, a 2.1 kbp promoter region of *OsIGI* was used to direct GUS expression in transgenic rice. As a result, GUS activity was mainly detected in young inflorescence (Fig. 1B), empty glumes and floret organs (Fig. 1C–E), such as in palea, lemma (Fig. 1C) and pistil (Fig. 1D) during early spikelet development stages. Additionally, GUS activity in the lodicules (Fig. 1E), filaments (Fig. 1E) and leaf (Fig. 1G) was observed as well. Furthermore, the elongating internode also showed a strong GUS signal in the transgenic plant driven by the *OsIG1* promoter (Fig. 1F), whereas very weak signal was detected in root (Fig. 1G). Overall, the expression patterns of *OsIG1* detected by qRT-PCR were consistent with the GUS signal shown in the transgenic lines.

To more precisely determine the spatial and temporal patterns of *OsIG1* expression in floral organs, we performed RNA *in situ* hybridization analysis. Strong signals of *OsIG1* were detected in the inflorescence meristems (Fig. 1H, I), floret primordia (Fig. 1J–L) and the empty glume (Fig. 1J–N). During organogenesis of florets, *OsIG1* transcripts were also detected in the primordia of stamens and ovules (Fig. 1L, M). Notably, *OsIG1* showed constitutive expression in nucellus (Fig. 1P) and later the detected signal was restricted to the micropylar end of the mature ovaries (Fig. 1Q). During embryogenesis, the *OsIG1* transcripts were detected throughout various stages of embryo development (Fig. 1R–T). As controls, no signals were detected in floral meristems and floral organ primordia with the sense probe (Fig. 1U, V).

**Fig. 2.**
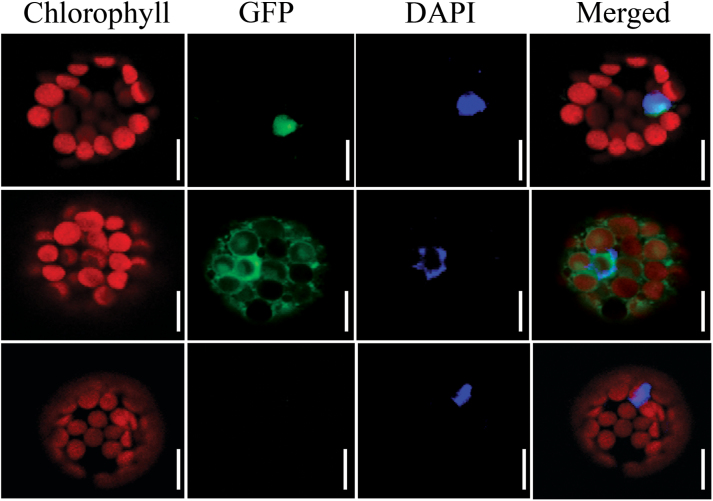
Localization of OsIG1-GFP fusion protein transiently expressed in *Arabidopsis* protoplasts. Upper panels, OsIG1-GFP; middle panels, 35S-GFP; bottom panels, an untransformed plant as a negative control. Left to right: red, chlorophyll autofluorescence; green, GFP fluorescence; blue, nucleus stained with DAPI; merged, combined fluorescence from GFP, chlorophyll and DAPI. Scale bars, 20 µm.

To determine experimentally subcellular localization, OsIG1 was fused in frame with GFP and transiently expressed in *Arabidopsis* mesophyll protoplasts. As expected, we found that the OsIG1-GFP fusion protein was unambiguously localized in the nucleus (Fig. 2), confirming that OsIGI is a nuclear protein.

### Alteration of floral development in *OsIG1*-RNAi repression plants

Because of the obvious expression of *OsIG1* in the floral tissues, we investigated the potential effects of *OsIG1* on rice floral development by constructing an inversely repeated RNAi vector (Supplementary Fig. 2A). Over 20 independent transgenic lines were obtained. Three representative transformants with 60–68% reduction of endogenous *OsIG1* expression level were selected for detailed phenotypic analysis (Supplementary Fig. 2B). To determine the specificity of *OsIG1* silencing, we searched the rice genomic sequence database Rice BLAST (http://RiceBLAST.dna.affrc.go.jp/) using the 485bp RNAi fragment, and found that four genomic loci (three loci located on *Os02g0318851* and one locus located on *Os05g34450*) with continuous 21–24bp short sequence identities to the RNAi fragment. The RNAi fragment showed 27% identity to *OsIAL1* (*Os05g0417000*) and 34% to *Os02g0318851*. Using qRT-PCR analysis we found that the expression pattern of the *OsIAL1* gene, with highest abundance in young inflorescence (Supplementary Fig. 3A), is similar to that of *OsIG1* (Fig. 1A). Subsequent measurement of expression levels for *Os05g0417000* and *Os02g0318851* revealed that their expression was nearly unaffected in the down-regulated *OsIG1* lines (Supplementary Fig. 3B, C). These results suggest there was only a low probability of incorrect targeting caused by *OsIG1-*RNAi and that the observed phenotypic alterations most likely resulted from down-regulation of *OsIG1* alone.

**Fig. 3.**
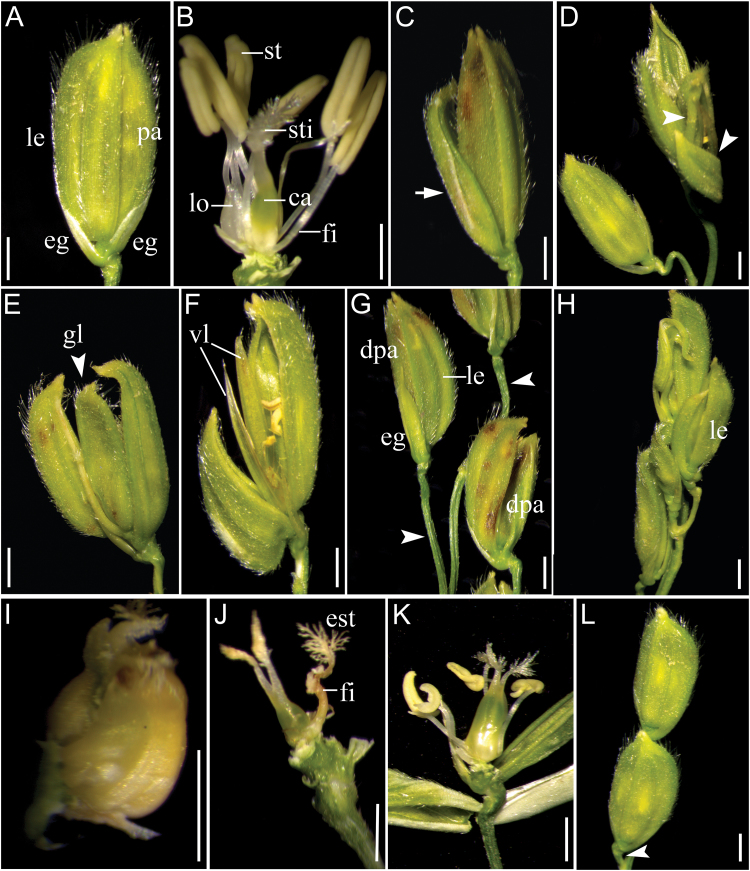
Phenotypes of WT and *OsIG1*-RNAi inflorescences and spiklets. (A, B) WT spiklet (A) and (B) with two lodicules, six stamens and one carpel. The two empty glumes, lemma and palea were removed for clarity in (B). (C–K) *OsIG1*-RNAi spiklet. The lemma and palea were removed in (I) and (J) for clarity. (C) A RNAi spikelet with elongated empty glume (arrow). (D) The number of empty glumes increased in the RNAi spiklet. (E) An extra glume-like organ formed in the RNAi spiklet. (F) A spikelet with velum-like organs. (G) A spikelet with degenerated or malformed palea in inflorescences at stage In9; arrowhead indicates peduncle. (H) The florets with severe phenotypes showing no inner floral organs. (I) Lodicules were fused at the base and transformed into carpel-like organs. (J) One stamen is transformed into a stigma-like organ in an *OsIG1*-RNAi spiklet. (K) The *OsIG1*-RNAi ovaries were clearly swollen compared with WT (B). (L) Inflorescences at stage In9 of WT; arrowhead indicates peduncle. ca, carpel; dpa, degenerated palea; eg, empty glume; est, ectopic stigma; fi, filament; gl, glume-like organ; le, lemma; lo, lodicule; pa, palea; st, stamen; sti, stigma; vl, velum-like organ. Scale bars, 1mm.

Next, we examined the effects of *OsIG1* deficiency on plant floral development in detail. Generally, a typical WT rice floret normally consists of a pistil in a central whorl (whorl 4), six stamens in whorl 3, two lodicules adjacent to the lemma in whorl 2 and two interlocking organs, palea and lemma, surrounding the inner floral organs in whorl 1. A floret with two pairs of sterile glumes (rudimentary glumes and empty glumes), which subtend at its base, constitutes a spikelet (Fig. 3A, B). Compared with WT, *OsIG1-*RNAi plants exhibited a wide variation in the number and position of floral organs as well as glumes (Fig. 3C–K). In the RNAi spikelets, empty glumes (also called sterile lemmas) were elongated and widened, resembling lemma/palea in shape and size (Fig. 3C). In addition to increased numbers of empty glumes (Fig. 3D) and extra glume-like organs (Fig. 3E), it was found that the formation of velum-like organs occurred in RNAi lines (Fig. 3F). In whorl 1, the paleae of the RNAi plants exhibited severe defects, including some smaller paleae (Fig. 3G) and others underdeveloped or distorted (Fig. 3H). In whorl 2, some lodicules were often fused at the base and transformed into carpel-like organs (Fig. 3I), suggesting that *OsIG1* may be required for lodicule specification in rice. In whorl 3, the filament-plumed stigma chimeras coupled with reduced stamen numbers were also visualized in approximately 5% spikelets of the RNAi lines (Fig. 3J). The *OsIG1*-RNAi ovaries (Fig. 3K) were clearly swollen compared to WT (Fig. 3B). Compared with WT, besides the abnormal morphology of floral organs, *OsIG1-*RNAi plants displayed altered inflorescence shape with elongated and curved peduncles (Fig. 3G, L).

**Fig. 4.**
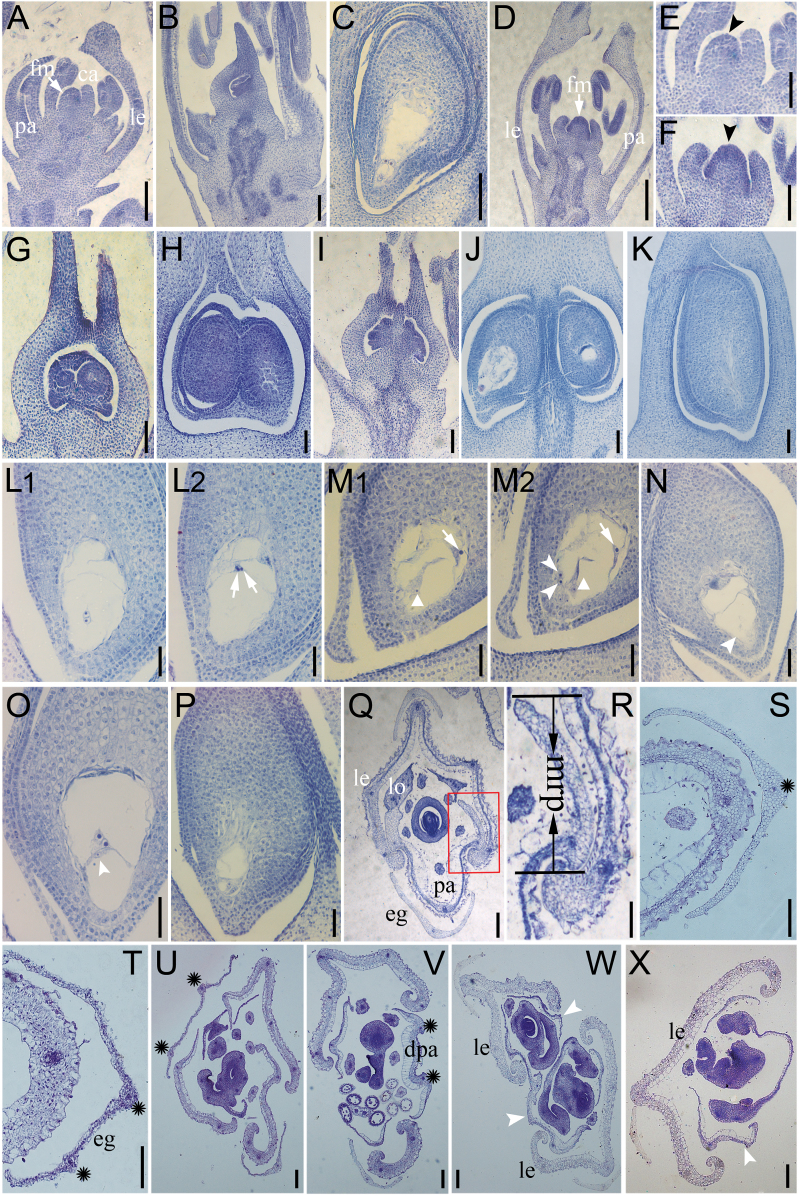
Histological analyses of WT and *OsIG1*-RNAi flowers. (A–C) Pistil development in the WT. A carpel protruded from the lemma side of the floral meristem (A). When the carpel completely enclosed the ovule primordium, a megaspore mother cell became visible and an integument primordium was differentiated in the ovule primordium (B). In a mature ovule, a vacuolated embryo sac was visible (C). (D) When a carpel protruded, the floral meristem in *OsIG1*-RNAi (D) was larger than that of the WT (A). (E, F) Close-up of floral meristem shown in the WT (A) and *OsIG1*-RNAi (D), respectively. (G–J) Unusual double ovules fused (G, H) or not fused (I, J) occurred in the transgenic ovaries. (K) Embryo sac degeneration in *OsIG1*-RNAi ovule. (L1, L2) Serial section showing *OsIG1*-RNAi embryo sac with extra polar nuclei (arrows). (M1, M2) Serial section showing *OsIG1*-RNAi embryo sac with possible multiple egg cells (arrowheads), extra synergids (triangular arrows) and polar nuclei located in an abnormal position close to the chalazal end (arrows). (N) Embryo sac without female germ unit (arrowhead). (O) Embryo sac with abnormal nuclear migration (or abnormal position of egg cell and synergid cells; arrowhead). (P) Abnormally small embryo sac. (Q–S) Cross-sections of a WT spikelet. (R) Magnification of the area within the box on Q showing interlocking of lemma and palea. (S) The WT empty glume with one vascular bundles (asterisk). (T-X) Cross-sections of the *OsIG1*-RNAi spikelets. (T, U) Two vascular bundles in the *OsIG1*-RNAi empty glumes (asterisks in T), as opposed to one in WT (asterisks in S). Edges of lemma and palea are hooked in the WT (Q), but there is no interlock with the lemma in the *OsIG1*-RNAi spikelet (U-X). (U) An abnormal flower with hull-like organs and two vascular bundles of empty glume (asterisks) in an *OsIG1*-RNAi flower. (V) The *OsIG1*-RNAi spikelet has only two full-developed vascular bundles (asterisks) in degenerated palea. (W) Transverse section of twin-flower spikelet (one spikelet containing two separate carpels) of *OsIG1*-RNAi; arrowheads indicate the degenerated palea. (X) The degenerated palea (arrowhead) lacking a bop and retaining mrp-like structures in an *OsIG1*-RNAi spikelet. ca, primary carpel; dpa, degenerated palea; eg, empty glume; fm, floral meristem; le, lemma; lo, lodicule; mrp, marginal region of palea; pa, palea. Scale bars, 100 μm (A–D, Q–X), 50 μm (E–P).

To characterize the observed morphological defect in more details, histological analyses were performed. In WT plants after the carpel primordium arises at the floral meristem near the lemma side, a lateral region of the floral meristem adjacent to the carpel initiation transformed into an ovule primordium (Fig. 4A) in which megaspore mother cell (Fig. 4B) occurs to form an embryo sac, the female gametophyte (Fig. 4C). By contrast, the size of the *OsIG1*-RNAi floral meristem at the early development stage of the pistil was larger than that of the WT (Fig. 4D–F). Also, the ovule primordia initiated on both the palea and the lemma side of floral meristem (Fig. 4D, G). Surprisingly, unusual double ovules, fused (Fig. 4G, H) or not fused (Fig. 4I, J), occurred in approximately 40% of the transgenic ovaries (Table 1). In addition, *OsIG1*-RNAi repression showed pleiotropic effects on female gametophyte differentiation, resulting in a variety of abnormally developed embryo sacs including embryo sac degeneration (Fig. 4K), an embryo sac with abnormal polar nuclei (Fig. 4L1, L2) and an embryo sac with an extra egg cell (Fig. 4M1, M2), as well as other abnormal embryo sacs, such as an embryo sac without a female germ unit (Fig. 4N), an embryo sac with abnormal nuclear migration (Fig. 4O) and an abnormally small embryo sac (Fig. 4P). The frequencies of various types of abnormalities are summarized in Table 1, with the RNAi lines showing 7–11% embryo sacs with abnormal polar nuclei, 27–29% embryo sac degeneration, 6–9% embryo sacs with an extra egg cell and 20–26% other abnormal embryo sacs.

**Table 1:** Frequencies of various types of abnormal ovules and embryo sacs in the *OsIG1*-RNAi repression lines (60 spikelets examined for each line were collected at the mature stage)

Genotype	One ovule					Double ovules					Total ovules examined
	ESD	EESWA	OAT	ESE	NES	ESD	ESWA	OAT	ESE	NES	
WT	0	0	1	0	59	0	0	0	0	0	60
RNAi-1	2	4	2	2	26	21	5	17	3	2	84
RNAi-2	3	3	2	3	28	19	3	14	4	2	81
RNAi-3	2	3	3	2	23	24	4	20	3	3	87

ESD, embryo sac degeneration; ESE, embryo sac with an extra egg cell; ESWA, embryo sac with abnormal polar nuclei; NES, normal embryo sac; OAT, other abnormal types.

Although the palea is similar to the lemma in its outer morphology in rice, a few critical differences exist: the lemma has five vascular bundles, whereas the palea has three vascular bundles and consists of two parts [the body of the palea (bop) and two marginal regions of the palea (mrps)]. The bop shares similar cellular morphology with lemma (Prasad *et al.*, 2005), but the mrp has a distinctive smooth epidermis specific to palea (Fig. 4Q, R; Chen *et al.*, 2006). In approximately 12% of the RNAi spikelets the elongated empty glumes had two vascular bundles as opposed to one in the WT (Fig. 4S–U). Interestingly, most of the degenerated palea (80%) exhibited reduced bop in size and nearly normal mrp (Fig. 4V). And about 6% degenerated palea lacked bop and retained only mrp-like structures with nonsilicified upper epidermis and without trichomes and protrusions (Fig. 4W, X), suggesting that the development of the bop of palea was severely affected in the RNAi lines. Additionally, all of RNAi lines displayed semi-sterile phenotype (the seed setting rate was approximately 38.6%).

To determine the nature of the reproductive defect found in the RNAi lines, we conducted reciprocal crosses and I_2_/KI staining assays for pollen from both WT and the RNAi lines. The results showed that offspring from the control cross (WT♀×WT♂) exhibited 86–91% seed-set fertility, while the pollination of WT pistils with pollen from RNAi plants produced seed-set rates ranging from 78 to 83% fertility. However, when using RNAi plants as the maternal recipient pollinated with WT pollen (RNAi♀×WT♂), the seed setting rates were scored as 46, 51 and 53% in *OsIG1*-RNAi repression panicles. Also, I_2_/KI assay showed that pollen from both WT (Supplementary Fig. 4A) and RNAi plants (Supplementary Fig. 4B–D) exhibited similar morphology and viability when fertilizing the emasculated rice spikelets. Taken together, these results suggest that the pollen from the RNAi plants was functional and that the sterility of the RNAi plants might be mainly due to defects in the female reproductive organs.

### RNAi plants exhibited defects in meristem maintenance as well as floral organ development

**Fig. 5.**
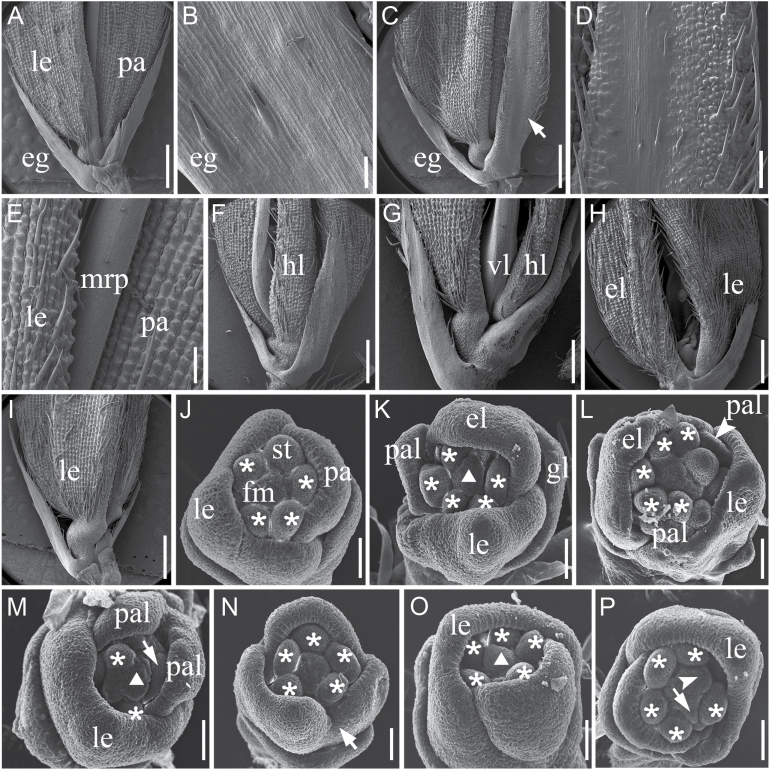
Scanning electron micrographs of WT and *OsIG1*-RNAi spikelets. (A) A WT spikelet. (B) Epidermal surface of an empty glume in the WT. (C) An *OsIG1*-RNAi spikelet with elongated empty glume (arrow). (D) Close-up view of the epidermal surface of the elongated empty glume in (C) showing a empty-glume-like surface in the central region and a lemma-/palea-like surface in the lateral region. (E) Epidermal surface of the lemma, palea and mrp in the WT. (F) An *OsIG1*-RNAi spikelet with an extra hull-like organ between the lemma and palea. (G) An *OsIG1*-RNAi spikelet with hull- or velum-like organs. (H) An *OsIG1*-RNAi spikelet with an extra lemma instead of the palea. (I) An *OsIG1-*RNAi spikelet with lacked palea. (J) WT flowers with six stamen primordia and a flat floral meristem. (K–P) *OsIG1*-RNAi flowers. (K) Showing one palea-like and one extra lemma primordium. (L) One abnormal flower with ectopic palea-like and extra lemma-like structures. (M) *OsIG1*-RNAi flower producing a stamen primordium ectopically in the centre of the floral meristem (triangular arrow), ectopic lodicule (arrow) and palea-like primordium. (N) One abnormal lemma shape. (O) At the late stage of stamen initiation, the floral meristem in *OsIG1*-RNAi lines remained bulged instead of being flat. (P) The floral meristem is uneven and bifurcated (arrow and arrowhead). eg, empty glume; el, extra lemma-like structure; fm, floral meristem; gl, glume-like organ; hl, hull-like organ; le, lemma; mrp, marginal regions of the palea; pa, palea; pal, palea-like organ; st, stamen; vl, velum-like organ. Asterisks indicate stamen primordia. Scale bars, 1mm (A, C, F–I), 100 µm (B), 200 µm (E, D), 50 µm (J–P).

To study the identity of the abnormal organs observed in the RNAi lines, we examined spikelets at the heading and flowering stage by scanning electron microscopy. Compared with the smooth outer epidermal surface of WT empty glumes (Fig. 5A, B), epidermal cells of the elongated empty glumes in the *OsIG1-*RNAi lines displayed bulges and bristles that are similar to phenotype of *eg1* mutation and ectopic expression of *OsMADS1* in rice (Fig. 5C, D; Jeon *et al.*, 2000; Prasad *et al.*, 2001; Li *et al.*, 2009). In *OsIG1-*RNAi lines, the extra hull-like organs showed a similar upper epidermis, resembling the WT lemma (Fig. 5E–G). In some cases, the palea was degenerated to form mrp-like structures (Fig. 5G). In other cases, an additional lemma-like organ occurred in place of *OsIG1*-RNAi palea (Fig. 5H), or *OsIG1-*RNAi spikelets lacked palea (Fig. 5I), suggesting that the development of palea was severely affected.

To further characterize the developmental defects of the *OsIG1*-RNAi spikelet, we investigated the flower morphology of down-regulated *OsIG1* transgenic lines at early stage by scanning electron microscopy. During the spikelet 6 stage (Sp6), the WT flower formed six stamen primordia, and it was apparent that the lemma with bumps at the top was larger than the palea (Fig. 5J). However, some *OsIG1*-RNAi spikelets developed extra lemma- or palea-like or glume-like structures (Fig. 5K–M). At the stage of stamen initiation, the floral meristem in WT flowers tended to be flat (Fig. 5J; Itoh *et al.*, 2005). By contrast, the floral meristem in RNAi lines still bulged (Fig. 5N, O), suggesting that floral meristem determinacy was severely affected. Occasionally, floral meristem was doubled in one spikelet (Fig. 5P), suggesting defects in floral meristem determinacy. Together these observations indicated that down-regulated *OsIG1* triggered abnormal spikelet development at an early stage, including the formation of ectopic floral organs, changes in organ number and alteration of floral meristem determinacy.

### Alteration of vegetative development in *OsIG1*-RNAi plants

**Fig. 6.**
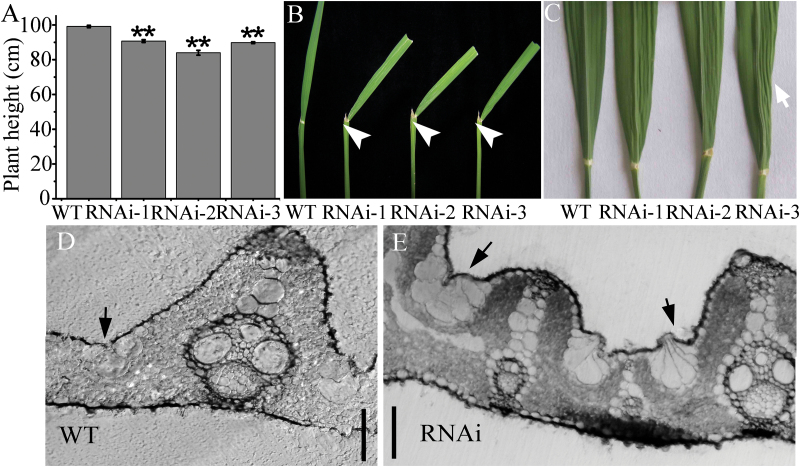
Vegetative phenotypic analyses of *OsIG1*-RNAi transgenic lines. (A) Plant height (cm). Student’s *t* test was used to analyse significant differences between WT and *OsIG1*-RNAi transgenic lines. Values are means ± SE from 20 plants. **P < 0.01 (*t* test). (B) Showing the increased leaf angle in *OsIG1*-RNAi transgenic lines. Arrows indicate the leaf lamina joint. (C) Some leaves of *OsIG1*-RNAi lines showing wrinkled blades, which was partially similar to that of plants overexpressing the class 1 *KNOX* gene *OSH1*. (D, E) Cross-sections of a WT (D) and *OsIG1*-RNAi (E) leaf blade.‍ Arrows indicate bulliform cells. Scale bars, 50 µm. A colour version of this figure is available at *JXB* online.

In addition to the altered morphology of reproductive organs, we also observed abnormal vegetative development by *OsIG1*-RNAi repression with reduced plant height and increased leaf inclination angle. Plant height for 20 field-grown individuals from each genotype was determined at the mature stage, and the average plant heights of the WT control, RNAi-1, RNAi-2 and RNAi-3 lines were 99.1, 90.7, 84.0 and 89.8cm, respectively (Fig. 6A), indicating a slightly reduced internodal elongation conferred by down-regulated *OsIG1* expression. Distinctly, an increased flag leaf inclination angle resulting from *OsIG1*-RNAi repression was visualized at the heading stage. Leaf angle is the angle of inclination between the leaf blade and the vertical culm (Zhao *et al.*, 2010). The lamina joint is a whitish region at the base of the blade, which joins the rice leaf blade and sheath and functions in bending the leaf blade toward the abaxial side. In the RNAi lines, the bending of the leaf blade occurred at the lamina joint. As shown in Fig. 6B, the leaf angle of the RNAi lines was much greater than that of the WT and the angle of leaves was increased and reached a maximum of approximately 44 °, whereas the leaf angle of WT plants appeared to plateau at approximately 16 °.

It has been reported that maize *ig1* mutant leaves have disrupted adaxial–abaxial polarity and fail to repress the expression of meristem-specific *KNOX* genes in leaf primordia, causing a proliferative, stem cell identity to persist in these cells (Evans, 2007). Occasionally, as shown in Fig. 6C, some leaves of *OsIG1*-RNAi lines exhibited wrinkled blades, resembling the phenotype observed in plants overexpressing the class 1 *KNOX* gene *OSH1* (Sato *et al.*, 1996), but no alteration of *OSH1* expression was detected in the leaves with the mutant phenotype in the *OsIG1*-RNAi lines (data not shown). Compared with the WT (Fig. 6D), the number of bulliform cells located between the vasculatures was significantly increased in the RNAi lines (Fig. 6E). In addition, these cells did not show a balloon-like form as occurring in the WT (Fig. 6E). No obvious difference in other leaf cell types or alteration in adaxial–abaxial polarity was observed between the RNAi lines and WT (Fig. 6D, E), indicating that the wrinkled blade phenotype may be caused by the increase in bulliform cell number. Together these results suggested that the *OsIG1* gene is involved in division and differentiation of bulliform cell and lateral growth during leaf development.

### Down-regulation of the *OsIG1* gene affects the expression of the *EG1*, *OsMADS6* and *OsMADS1* genes

**Fig. 7.**
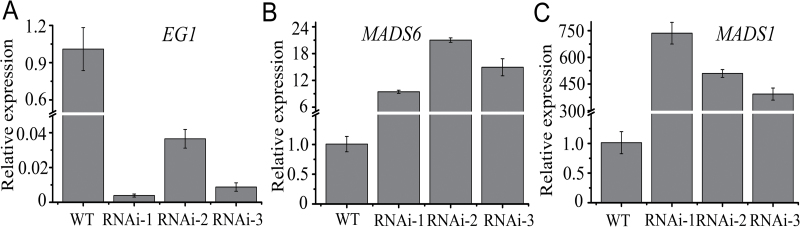
Comparison of gene expression levels of *EG1*, *OsMADS1* and *OsMADS6* in the inflorescences of *OsIG1*-RNAi transgenic lines and WT. qRT-PCR analysis of *EG1*, *OsMADS1* and *OsMADS6* transcripts in 5cm inflorescences in the WT and *OsIG1*-RNAi lines. *Actin* expression in each sample was used as a control. The expression level in the WT was set as 1. Error bars represent SD from three independent biological replicates.

Recently, a number of plant genes involved in floral organ development have been identified. We asked whether the *OsIG1* gene mediates floral development by regulating well-characterized floral-organ-associated genes. To this end, we investigated the expression of several genes which are profoundly involved in this process, including putative B function genes (*OsMADS2* and *OsMADS16*), C function genes (*OsMADS3*, *OsMADS58*), a *YABBY* gene *DROOPING LEAF* (*DL*), a D function gene (*OsMADS13*), an E function gene (*OsMADS1*), an *AGAMOUS*-*LIKE6* (*AGL6*)-like gene (*OsMADS6*) and *KNOX* genes (*OSH1* and *OSH6*), as well as CLV1-homologue *FON1* and jasmonic acid-synthesizing gene *EG1* in the inflorescences of WT and the *OsIG1*-RNAi lines (less than 1, 5, 10, 15 and 20cm in length) using qRT-PCR analyses (Agrawal *et al.*, 2005; Cai *et al.*, 2014; Dreni *et al.*, 2007; Jeon *et al.*, 2000; Kater *et al.*, 2006; Kyozuka *et al.*, 2000; Li *et al.*, 2009, 2010; Matsuoka *et al.*, 1993; Moon *et al.*, 1999; Nagasawa *et al.*, 2003; Ohmori *et al.*, 2009; Suzaki *et al.*, 2004; Yamaguchi *et al.*, 2004, 2006; Zhang *et al.*, 2013). The results showed that in *OsIG1*-RNAi lines *EG1* expression was dramatically reduced by 62-fold (Fig. 7A), while expression of *OsMADS6* and *OsMADS1* was up-regulated by 15- and 545-fold compared with the WT (Fig. 7B, C), respectively. Other than this, we did not observe consistent changes in the expression of other genes tested among the transgenic and WT plants (Supplementary Fig. 5). These results suggested that *OsIG1* regulates the morphogenesis and development of floral organs, probably by regulating the expression of *EG1* and the floral organ identity genes *OsMADS1* and *OsMADS6* as well as by unidentified pathways.

## Discussion

### OsIG1 may function to maintain empty-glume identity

Regarding the origin and evolution of the sterile lemma, there are two controversial hypotheses. One hypothesis considers that an ancestral rice spikelet has three florets, including one fertile floret and the other two degenerated sterile florets with only the lemmas remaining (Kellogg, 2009). The empty glumes thus would seem to be just the remnants of the two lower reduced florets (Yoshida *et al.*, 2009; Kobayashi *et al.*, 2010). Another hypothesis states that the rice spikelet has only one floret, and the sterile lemma and rudimentary glume are generally regarded as severely metamorphic bract structures (Schmidt and Ambrose, 1998; Terrell *et al.*, 2001; Hong *et al.*, 2010). Recently, several mutants and their underlying genes related to the development of glume-like organs have been identified in rice, including *eg1* (Li *et al.*, 2009), *long sterile lemma* (*g1*; Yoshida *et al.*, 2009)/*elongated empty glume1* (*ele1*; Hong *et al.*, 2010) and *osmads34* (Gao *et al.*, 2010). Among these, *g1*/*ele1* and *osmads34* mutants displayed the transformation of sterile lemmas into lemma-like structures (Yoshida *et al.*, 2009; Hong *et al.*, 2010; Gao *et al.*, 2010), suggesting that *G1*/*ELE1* and *OsMADS34* repress lemma identity at the sterile lemma positions during rice spikelet development (Yoshida *et al.*, 2009; Hong *et al.*, 2010). Unlike *g1*/*ele1* and *osmads34* mutants, *eg1* mutation severely affects its number as well as sterile lemmas size (Cai *et al.*, 2014). These observations suggest that different genes are probably involved in the pathway of empty-glume development. In our *OsIG1*-RNAi lines, we observed an increased number of empty glumes, and in some cases the empty glumes were converted into glume-like organs and the increased number of empty glumes occurred (Fig. 3D). Similar to the studies on *EG1*, *OSMADS34* and *G1*/*ELE1*, our data for *OsIG1* seems supporting the first hypothesis.

Additionally, nuclear localization of IG1 and its expression at the early developmental stage of empty glumes suggests that IG1 may achieve this function by targeting downstream genes needed for empty-glume growth. As in the loss-of-function *eg1* mutation, the spikelets of *OsIG1*-RNAi lines developed extra glume-like structures (Fig. 3E; Li *et al.*, 2009). In addition, in *OsIG1*-RNAi lines empty glumes were elongated and widened, resembling the lemma/palea in shape and size, a phenotype similar to that reported by Prasad *et al*. (2001, 2005), where *OsMADS1* over-expression created a lemma-like epidermis in the transformed glumes. Intriguingly, these phenotypic alterations appear to be consistent with *EG1* down-regulation and *OsMADS1* up-regulation observed in *OsIG1*-RNAi repression lines (Fig. 7A, C). Our data showed that *OsIG1*-RNAi repression (by 35 S promoter) could greatly enhance expression of *OsMADS6* and *OsMADS1* (Fig. 7B, C), and we believe that the abnormal organ development occurring in *OsIG1*-RNAi lines could be ascribed to, at least in part, the constitutive/ectopic expression of these *MADS* genes (Fig. 3C, I, J). Also, our qRT-PCR analysis showed that the expression level of *EG1* was remarkably decreased in all of three *OsIG1*-RNAi repression lines relative to WT (Fig. 7A), indicating that EG1 might function downstream of OsIG1. Nevertheless, Li *et al.* (2009) and Cai *et al.* (2014) reported that EG1 positively regulates expression of the floral meristem and organ identity gene *OsMADS1*, seemingly contradictory to our expression data for *EG1* and *OsMADS1* in the *OsIG1*-RNAi lines. However, this discrepancy implicates the complexity of floral organ gene expression and regulation under various concerts of background. Actually, Cai *et al.* (2014) observed a significant reduction of *OsMADS1* expression in 2mm inflorescence of *EG1*-defective lines, but a large portion of this reduction disappeared in the 3–4mm inflorescence, suggesting their interaction is spatially and temporarily regulated. Different from these observations, opposite expression alteration of *OsMADS1* and *EG1* was visualized in the background of *OsIG1*-RNAi lines (Fig. 7A, C). We thought it is probably the different background that should be responsible for triggering the unusual activation/repression of gene expression. Although OsMADS1 has been shown as a positive target of EG1, it can not be ruled out that OsMADS1 acts as a target protein directly or indirectly regulated by other regulatory proteins, such as OsIG1 in the current study or other unidentified regulators. Therefore, our results point out that the effects of *OsIG1*-RNAi repression are probably not limited to EG1 function and that *OsMADS1* as well as its unknown upstream genes also could be activated/repressed in an EG1-independent pathway in *OsIG1*-RNAi lines. It is indeed interesting that down-regulation of *OsIG1* results in up-regulation of *OsMADS1* expression in these RNAi lines, suggesting that there may be interplay between OsIG1 and the MADS box to mediate certain developmental cascades in an EG1-independent pathway. Nevertheless, further investigations are required to examine whether this interaction genuinely exists in the regulation of rice floral development. In addition, it is worth mentioning that another characteristic phenotype of the *OsIG1*-RNAi lines—that is, double ovules—does not occur in loss-of-function *eg1* mutant (Li *et al.*, 2009; Cai *et al.*, 2014), suggesting that unidentified downstream genes of *OsIG1* other than *EG1* could be responsible for the initiation of ovule number control. All these hypotheses need to be further tested in the future.

### OsIG1 regulates palea development

At present, several important genes are known to be involved in specifying the identity of the palea. In the *mfo1* mutant mrp is absent and recent studies revealed that *MFO1*/*OsMADS6* is expressed in the mrp (Ohmori *et al.*, 2009; Li *et al.*, 2010). Like the *mfo1* mutant, in RNAi lines of *LHS1*/*OsMADS1*, mrp is absent in the abnormal palea (Prasad *et al.*, 2005). These results imply that *OsMADS1* and *OsMADS6* specify palea identity by promoting mrp development. In contrast, *dp1*, *sdp1*/*rep1* and *osmads15* mutants all exhibited changes in the bop rather than the mrp (Li *et al.*, 2008; Wang *et al.*, 2010; Yuan *et al.*, 2009). Our findings show that in most of the degenerated palea the development of mrp was normal while the size of bop was reduced in *OsIG1*-deficient lines in which the E function gene *OsMADS1* and the functionally similar E function gene *OsMADS6* were up-regulated. Apparently, the altered expression levels of *EG1*, *OsMADS1* and *OsMADS6* in *OsIG1*-RNAi lines contributed to the abnormal formation of the palea. Since the bop tissue alone was affected by *OsIG1*-RNAi repression, it is possible that the bop and mrp of the palea are controlled by different pathways. Indeed, several genes, including *DP1*, *SDP1*/*REP1* and *OsMADS15*, have been reported to determine bop identity (Li *et al.*, 2008; Yuan *et al.*, 2009; Wang *et al.*, 2010). Furthermore, *LHS1*/*MADS1* and *MFO1*/*MADS6* have been demonstrated to be involved in the regulation of mrp identity (Prasad *et al.*, 2005; Ohmori *et al.*, 2009). Additionally, *DEGENRATED HULL1* (*DH1*), a LOB family transcription factor gene, was found to affect both palea and lemma formation (Li *et al.*, 2008; Wang *et al.*, 2011). Thus, it is possible that various genes, such as E function-like genes (*OsMADS1* and *OsMADS6*), palea-specific genes (*OsMADS15*, *DP1* and *SDP1*/*REP1*), as well as the *LBD* gene *OsIG1* are required for the formation of palea.

### OsIG1 is required for patterning and growth during ovule development

In the maize *ig1* mutant, embryo sacs undergo extra rounds of free nuclear division that result in extra egg cells, extra central cells and extra polar nuclei, and also the mutation shows disrupted adaxial–abaxial polarity of leaf development (Huck *et al.*, 2003). Our work revealed that the down-regulated *OsIG1* had a similar disruption to embryo sac development (Table 1, Fig. 4L–P), but did not obviously influence the leaf adaxial–abaxial patterning as in the maize *ig1* mutant (Evans, 2007), in spite of the altered inclination angle observed in the RNAi transgenic flag leaves (Fig. 6B). The phenotypic variations might be associated with unchanged meristem-specific *KNOX* gene expression between the transgenic rice lines and WT (data not shown), compared to the fact that alteration of *KNOX* gene expression is associated with adaxial–abaxial polarity in maize (Evans, 2007). Alternatively, it is possible that the rice RNAi lines retaining relative high residual *OsIG1* transcript activities compared with the maize *ig1* loss-of-function mutant might be the cause of the unaffected leaf adaxial–abaxial patterning. Interestingly, besides glume-like features, other unique characteristics occurring in *OsIG1*-RNAi lines has not yet been reported in rice *eg1* and maize *ig1* mutants, including abnormal carpels and unusual fused double ovules, suggesting that *IG1* functions have diversified between the two species. Although our experiments suggest there is less possibility of incorrect targeting caused by *OsIG1-*RNAi, we could not completely exclude the possibility that the potential incorrect targeting might have effects on vegetative and reproductive processes. Hence, specific mutated lines (e.g. T-DNA insertion lines) would be a useful tool to confirm the phenotypic abnormalities triggered by *OsIG1-*RNAi repression.

Meanwhile, alteration of floral organ number has been demonstrated to be closely associated with a change in meristem size (Suzaki *et al.*, 2004). In this study, silencing *OsIG1* increased floral meristem size at the stage of carpel protrusion (Fig. 4D, F), resulting in increased ovule number (Fig. 4F, G). Additionally, ovule development was indistinguishable from that of the WT at a very early developmental stage. As shown in Fig. 4G, the change in ovule primordia size was accompanied by shape modification, evidenced by the unusual ovule primordia initiation existing on both sides of the palea and lemma of the floral meristem (Fig. 4D, G). This observation raises the possibility that the initiation of ovule primordia is disrupted by *OsIG1*-RNAi repression. Therefore, the OsIG1 protein may play an important role in inhibiting ovule primordium formation on the palea side in rice. Although it is well known that establishment of adaxial–abaxial polarity is essential for lateral organ development, little is known about the mechanisms underlying the polarity establishment in the carpel and ovule. On the basis of these observations, we speculate that although adaxial–abaxial polarity was less affected in the leaves of *OsIG1*-RNAi plants, the unusual ovule patterning of transgenic lines is presumably due to defects in the regulation of polarity. The polarity of the gynoecium (the lemmal versus paleal side) may be lost and the gynoecium forms two ovules instead of one. We propose that the gynoecium might contain two ovaries if the transformation is complete (Fig. 4H, I); however, incomplete transformation may result in the fused ovule phenotype (Fig. 4G, H).

In addition, given that aberrant shapes of ovule primordia were initiated by down-regulation of *OsIG1* (Supplementary Fig. 2B), we postulated that *OsIG1* might have an important role in maintaining the proper organization of the floral meristem, which is essential for the initiation of ovule primordium. In other words, *OsIG1* may regulate carpel and ovule primordium initiation by maintaining the proper floral meristem organization during the early developmental phase in rice. Despite the fact that an increased size of the floral meristem/ovule primordial occurred in *OsIG1*-RNAi repression, the key genes which regulate ovule primordial initiation need to be identified. On the other hand, in the maize *ig1* mutant, the proliferative phase is prolonged, suggesting that the IG1 protein restricts the proliferative phase of female gametophyte development. According to this opinion, it is possible that *OsIG1* regulates carpel and ovule numbers by preventing overproliferation of their primordia in rice.

In this study, our results revealed that *OsIG1* acts as a key regulator in controlling rice spikelet development. It was reported that *IG1* interacts with *ROUGH SHEATH2* (*RS2*), which is the maize orthologue of *AtAS1* (Evans, 2009). Furthermore, it was shown that members of the basic helix-loop-helix (bHLH) family of transcription factors are capable of interacting with LOB (Husbands *et al.*, 2007). Thus, it would be interesting to elucidate whether these genes are the targets of *OsIG1* that may be involved in ovule development. Future identification of the specific interacting factors will shed new light on the molecular mechanism of ovule development in grasses.

## Supplementary data

Supplementary material is available at *JXB* online.

Supplementary Fig. 1. Structure of *OsIG1* and sequence analysis of *OsIG1*.

Supplementary Fig. 2. Constructs and molecular analysis of normal and transgenic plants.

Supplementary Fig. 3. Expression patterns of *OsIAL1* and the expression levels of *OsIAL1* and *Os02g0318851* in the young panicles of *OsIG1*-RNAi transgenic lines and WT plants.

Supplementary Fig. 4. The I_2_/KI staining of pollen grains of the WT (A) and *OsIG1*-RNAi transgenic lines (B–D).

Supplementary Fig. 5. Expression-level comparison of flower-development-related genes between WT and *OsIG1*-RNAi lines in 1, 5, 10, 15 and 20cm inflorescences.

Supplementary Table 1. Primers used in this study.

Supplementary Data

## Abbreviations:

bop: body of the palea
bp: base pairs
GFP: green fluorescent protein
GUS: β-glucuronidase
LBD: LATERAL ORGAN BOUNDARIES DOMAIN/ASYMMETRIC LEAVES2-LIKE
LOB: LATERAL ORGAN BOUNDARIES
mrp: marginal regions of the palea
qRT-PCR: real-time quantitative PCR
WT: wild type.
