# Supplementary material for: Down-regulation of a LBD-like gene, OsIG1, leads to occurrence of unusual double ovules and developmental abnormalities of various floral organs and megagametophyte in rice
Source: J Exp Bot. 2014 Oct 16;66(1):99–112. doi: 10.1093/jxb/eru396 (PMC4265153; doi:10.1093/jxb/eru396)
Supplement: Supplementary Data [file supp_66_1_99__index.html]

Down-regulation of a LBD-like gene, OsIG1, leads to occurrence of unusual double ovules and developmental abnormalities of various floral organs and megagametophyte in rice — Down-regulation of a LBD-like gene, OsIG1, leads to occurrence of unusual double ovules and developmental abnormalities of various floral organs and megagametophyte in rice — Supplementary Data 

# Down-regulation of a *LBD*-like gene, *OsIG1*, leads to occurrence of unusual double ovules and developmental abnormalities of various floral organs and megagametophyte in rice

## Supplementary Data

Data files

**Files in this Data Supplement:**

- Supplementary Data - Supplementary Data
