# Supplementary material for: Down-regulation of a LBD-like gene, OsIG1, leads to occurrence of unusual double ovules and developmental abnormalities of various floral organs and megagametophyte in rice
Source: J Exp Bot. 2014 Oct 16;66(1):99–112. doi: 10.1093/jxb/eru396 (PMC4265153; doi:10.1093/jxb/eru396)
Supplement: Supplementary Data [file supp_eru396_jexbot126953_file001.pdf]

Supplementary Data

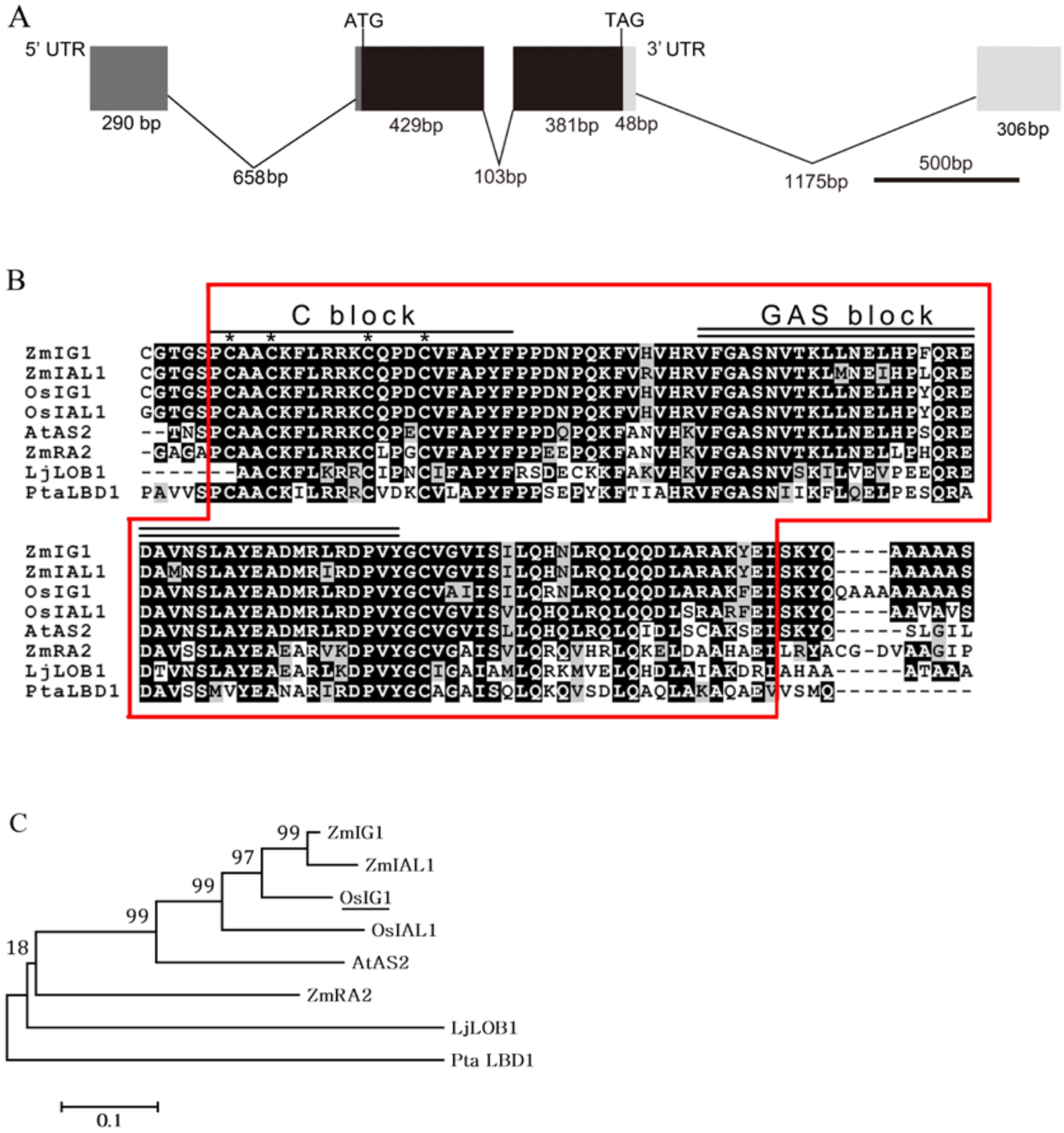

Fig. S1. Structure of *OsIG1* and sequence analysis of *OsIG1*.

(A) Structure of *OsIG1* gene. Black boxes indicate exons and thick lines indicate introns. The coding regions are shown by black boxes.

(B) Domain scanning of OsIG1. The deduced amino acid sequences of the OsIG1 gene were aligned with previously reported genes from other species using the clustal W version 1.82. The red boxed region is LOB-domain, and conserved C blocks and GAS blocks were underlined with single and double lines, respectively. The cysteine residues in the C-motif are shown with asterisks.

(C) Phylogenetic analysis of OsIG1 and LBD proteins of other plants. The phylogenetic tree was constructed using MEGA4 via the neighbor-joining method. Bootstrap values from 1000 replicates were calculated and are indicated at branch points on the neighbor-joining tree. The tree includes ZmIG1(EF081454), ZmIAL1(EF081455), ZmRA2 (DQ327701) from *Zea mays*, AS2(AB080802) from *Arabidopsis thaliana*, LjLOB1 (AY790249) from *Lotus japonicus*, PtaLBD1（HQ284165）from *Populus tremula* and OsIAL1（TC298324）from *Oryza sativa*.

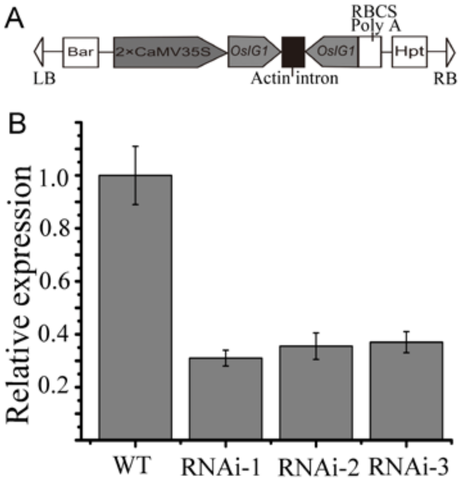

Fig. S2. Construct and molecular analysis of normal and transgenic plants.

(A) Schematic diagram of part of the T-DNA region of the transforming construct CMV35S-*OsIG1*-RNAi. Inversely repeated fragments derived from 3’ coding region and UTR of *OsIG1* are indicated by *OsIG1*.

(B) Real-time quantitative RT-PCR analysis of *OsIG1* mRNA levels in flowers derived from wild type (WT) and three independent transgenic lines. Each bar represents three replications from each RNA sample. Error bars represent standard errors shown in each case.

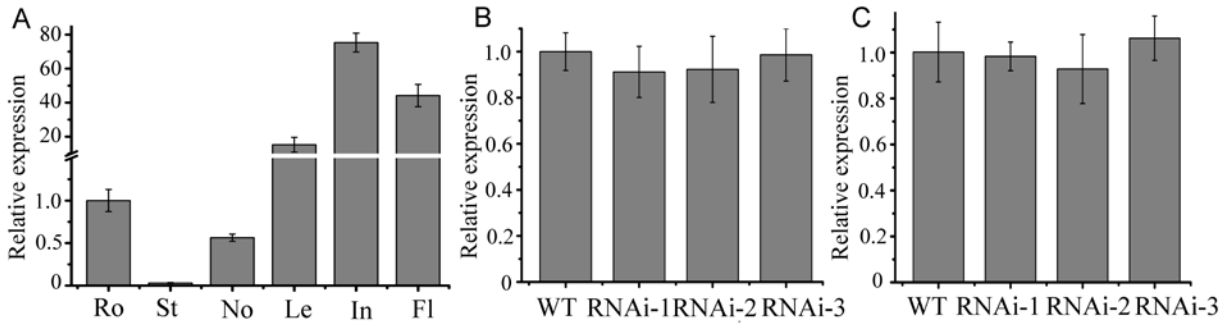

Fig. S3. Expression patterns of *OsIAL1* and the expression levels of *OsIAL1* and *Os02g0318851* in the young panicles of *OsIG1*-RNAi transgenic lines and wild type. For comparison the expression level in the wild type was set as 1. Error bars represent ± SD from three independent biological replications.

(A) Expression patterns of *OsIAL1*in wild type

(B) qRT-PCR analysis of expression levels of *OsIAL1* in young panicles of wild type and *OsIG1*-RNAi lines.

(C) qRT-PCR analysis of expression levels of *Os02g0318851* in young panicles of wild type and *OsIG1*-RNAi lines.

Fl, mature flower; In, young inflorescence; Le, leaf; No, node; Ro, roor; St, stem.

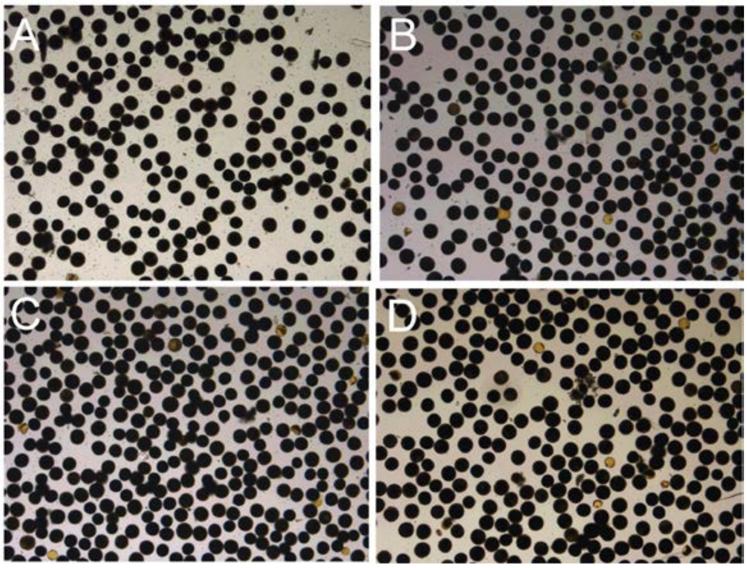

Fig. S4. The I_2-KI staining pollen grains of the wild type (A) and *OsIG1*-RNAi transgenic lines (B)-(D).

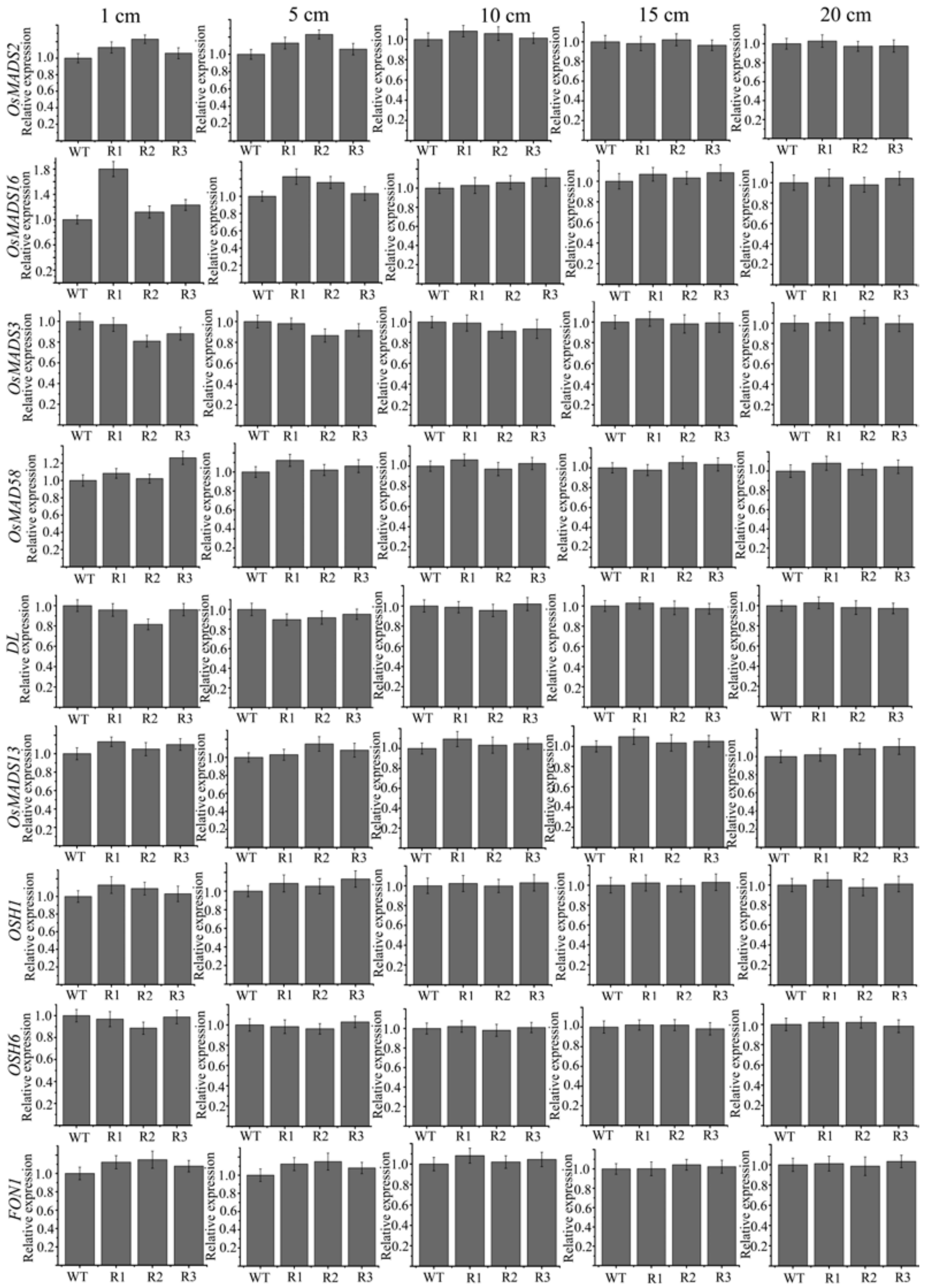

Fig. S5. Expression level comparison of flower development related genes between wild type and *OsIG1*-RNAi lines in 1cm, 5cm, 10 cm, 15cm, 20 inflorescences.

FON1, *FLORAL ORGAN NUMBER1*; *DL*, *DROOPING LEAF*; R1, RNAi-1 line; R2, RNAi-2 line; R3, RNAi-3 line.

Error bars represent ± SD from three independent biological replications.

Table S1. Primers were used in this study.

Primers used in the RNAi constructs of the *OsIG1*gene		
Primer name	Primer sequence	Enzyme site
IG1IF1	5'-CCGCTCGAGCTGCAGACGGGGACGAATAACGG-3'	*Xho*I, *Pst*I
IG1IR1	5'-CCCAAGCTTGCGAGCAATCCTCCTAAGC-3'	*Hind*III
IG1IF2	5'-TGCTCTAGAACGGGGACGAATAACGG-3'	*Xba*I
IG1IR2	5'-CGCGGATCCGCGAGCAATCCTCCTAAGC-3'	*Bam*HI
**Primers used for RT- PCR analysis**		
Primer name	Primer sequence	Gene name
IG1-F	5'-TCATCAACGTCGGGCACTC-3'	*IG1*
IG1-R	5'-GAGACGAACACAACAACCGC-3'	
HPT-F	5'-TAGGAGGGCGTGGATATGTC-3'	*HPT*
HPT-R	5'-TACACAGCCATCGGTCCAGA-3'	
ACTIN-F	5'-AAGATCCTGACGGAGCGTGGTTAC-3'	*ACTIN*
ACTIN-R	5'-CTTCCTAATATCCACGTCGCACTTC-3'	
IAL1-F	5'-TGAGGAGAATAACCAAGAGC-3'	*IAL1*
IAL1-R	5'-TGTAACAACACATTTTCTTCTG-3'	
**Primers used for quantitative real-time RT- PCR analysis**		
Primer name	Primer sequence	Gene name
q*UBQ*-F	5'-CACCCTGGCTGACTACAACA-3'	*UBQ*
q*UBQ*-R	5'-TTCTTCTTGCGGCAGTTGAC-3'	
q*IG1*-F	5'-TTCATCAACGTCGGGCACT-3'	*IG1*
q*IG1*-R	5'-CTCCCCTTCGTAGCTCCTC-3'	
q*DL*-F	5'-CAATGGATCTCGTGTCG-3'	*DL*
q*DL*-R	5'-TGAAGCGGTTGTAAGCAG-3'	
q*ACT*-F	5'-CGTATGAGCAAGGAGATCAC-3'	*ACTIN*
q*ACT*-R	5'-CACATCTGTTGGAAGGTGCT-3'	
qOs1-F	5'-ATCACCATCAGGGTCTTCTC-3'	*OsMADS1*
qOs1-R	5'-CAACCATGTCTGCTGCTTCA-3'	
qOs2-F	5'-CAGCAAGATATAGCGCTGAG-3'	*OsMADS2*
qOs2-R	5'-ATTGTTCTCCTGCAGGTTGG-3'	
qOs3-F	5'-GACAGCAGCCACTGAACATG-3'	*OsMADS3*
qOs3-R	5'-AGCTGATGGGCGTAATGCTG-3'	
qOs6-F	5'-AGAGAAAGACGCAACTGATGATGG-3'	*OsMADS6*
qOs6-R	5'-AGGCTTGCTGCATGGCTCTG-3'	
qOs13-F	5'-ATGGGGAGGGGCAGGATTGAG-3'	*OsMADS13*
qOs13-R	5'-TGCGCCTTCTTGTACCTGTCA-3'	
qOs58-F	5'-GAGCAAAGTTGCTGAGAGTG-3'	*OsMADS58*
qOs58-R	5'-GAGGCTGATGCATGATGTTG-3'	

q*EG1*-F	5'-AACGTACACGACCCGATCAC-3'	*EG1*
q*EG1*-R	5'-GACGTGGGTGTAGCAGGAGT-3'	
q*OSH6*-F	5'-CGGTCGGCACTGCTTGA-3'	*OSH6*
q*OSH6*-R	5'-CAGCTTATCTTCTTCCGTGGGATA-3'	
q*OSH1*-F	5'-GCTCAACACGCTCTCCATCTC-3'	*OSH1*
q*OSH1*-R	5'-GTGCATCAATCTCAGGTAGCTCTGT-3'	
q*FON*-F	5'-CGTCAAGTCCAACAACATCC-3'	*FON1*
q*FON*-R	5'-AATAGCACCACACCGAAGC-3'	
**Primers used in the constructs for *OsIG1* promoter :: GUS fusion construct**		
Primer name	Primer sequence	Enzyme site
IGPF	5’-CCGGAATTCTCCTCTGTCCATTCTCAAATAACT-3’	*Eco*RI
IGPR	5’-CCCAAGCTTTCCTCCTCGCCTGGGAAAG-3’	*Bam*HI
**Primers used in the constructs for OsIG1-GFP fusion construct**		
Primer name	Primer sequence	Enzyme site
IG1::GFP-F	5’-CCCTCGAG ATGGCGTCATCGTCAGCGT-3’	*Xho* I
IG1::GFP-R	5’-GGAATTCCTGGCCGGCGCCTTGCCT-3’	*Eco*RI
**Probe preparation for *IG1***		
OsIG1 probe-F	5’-TCATCAACGTCGGGCACTC-3’	*OsIG1*
OsIG1 probe-R	5’ -GAGACGAACACAACAACCGC-3’	
